# Toward Planetary Health Ethics? Refiguring Bios in Bioethics

**DOI:** 10.1007/s11673-023-10285-0

**Published:** 2023-08-25

**Authors:** Warwick Anderson

**Affiliations:** Anthropology, SSPS, Social Science A02, University of Sydney, Sydney, NSW 2006 Australia

**Keywords:** Bioethics, Planetary health, Disease ecology, Crisis, COVID-19, History

## Abstract

In responding to perceived crises—such as the COVID-19 pandemic—in routinized ways, contemporary bioethics can make us prisoners of the proximate. Rather, we need bioethics to recognize and engage with complex configurations of global ecosystem degradation and collapse, thereby showing us paths toward co-inhabiting the planet securely and sustainably. Such a planetary health ethics might draw rewardingly on Indigenous knowledge practices or Indigenous philosophical ecologies. It will require ethicists, with other health professionals, to step up and become public advocates for environmental sustainability. The COVID-19 pandemic should be seen as opening a portal to planetary health ethics or ecologized bioethics.

One night, in the gloom of another COVID-19 lockdown, I believe I saw some rough beast slouching in the general direction of Hygeia. A revelation of renewed bioethics, a redemptive formation, is perhaps at hand.

Mind you, the pandemic from the start has already generated copious bioethical inquiry, richly fertilized by talk of crises and emergencies. The moment we define or perceive an event as a crisis, we are forced into quick and frequent moral judgement, often hastily apprehended, before finding time for adequate reflection and appraisal (Roitman 2014; Anderson 2021a). As Paul Komesaroff and colleagues (2020, 461) put it: “The unfolding crisis has imposed a need on many people to make decisions with deep, sometimes unprecedented, ethical content.” It seems we must do so despite scanty information, contested facts, and distorted speculations. “The COVID crisis, or any crisis,” writes Paul James (2020, 489), “is not a good time for developing ethical precepts on the run…. Such reactive ethics tends to lead to either individualized struggles over the right way to act or hasty sets of guidelines that leave out contextualizing questions concerning regimes of care.” A cynical reader may interpret that last statement to mean that a crisis precipitates a deluge of the usual ethical formulations, just more of the usual stuff, retooled. Thus, we get important yet predictable accounts of the ethical challenges of social distancing, masking, confidentiality, stigmatization, discrimination, lockdowns, border closures, arbitrary detention, resource allocation, vaccine mandates, and so on. Ethics discourse of this kind is necessary and compelling, of course, but hardly unprecedented. Too often, the bioethics of first resort lacks even the novelty of the now not so novel coronavirus.

And yet, Komesaroff (2020, 516) tells us: “the disruption itself can open the way to an unexpected, even welcome, destabilization of old assumptions, habit, expectations, and values.” Or as James (2020, 489) asks insistently: “What does it meant to develop an ethics in response to the COVID crisis?” That is, how might we discern ethically situated and responsible bioethics, renewed and reinvigorated bioethics, amongst all this mess? This is the subject I want to consider here.

## Configuring Theory in a Pandemic

For some of us, how to theorize under duress is an old question, predating the current pandemic. We felt the same pressing demand in the 1980s during the AIDS pandemic—at the time another “crisis” causing us to take stock of our lives and our places in the world. The endeavour then to understand and properly inhabit our troubled condition often found expression in the striving for “theory” in an epidemic. For Paula Treichler (1999, 9), the critical response to AIDS was “the struggle for an intelligent vision to live by in the face of crisis, contradiction, and the urgent need to make life-or-death decisions”—not a bad description, surely, of what bioethical inquiry should be in response to COVID-19. Since the late 1980s, Treichler had been pleading for “the careful examination of language and culture that enables us, as members of intersecting social constellations, to think carefully about ideas in the midst of a crisis” (1). Initially, discussion of the ethical responses to AIDS had focused on the usual suspects: problems of discrimination and stigmatization, concerns about confidentiality and privacy, debates over the steps needed to limit contagion (Kelly 1987; Schuklenk 2001). As time wore on, as the temporal frame shifted from epidemic crisis to endemic “normality,” to the time when Treichler began writing, a different style of ethics analysis emerged. In developed countries, those communities most affected by HIV campaigned for greater resources to combat the disease; they challenged standard methods of testing and accessing preventive measures and new treatments; they tried to seize control of the methods of clinical research and the ethics of care (Epstein 1996). In the 1990s, too, the spread of AIDS across the world prompted concerns about exploitation of research subjects and access to antiretrovirals in poorer countries, giving rise to what became known as “global health,” a biosecurity enterprise with ethical predicates (Farmer 2001; Brandt 2013; Packard 2016). And so, sometimes subtly, sometimes bluntly, the paradigmatic pandemic of the late-twentieth century eventually transformed ethical conversations.

The emergence of HIV, a novel virus after all, in the 1980s also began directing the attention of some elite biomedical scientists toward ecological and evolutionary dynamics determining the distribution and abundance of viruses and other pathogenic agents (Morse 1993). They turned to an older, though often marginalized and neglected, tradition of disease ecology, to earlier inquiries into the interactions of animal hosts and their “parasites,” which include bacteria and viruses (Anderson 2004). These ecologically minded medical researchers wanted to know how infectious disease might emerge and decline in human populations in conformation with environmental disruption, social change, and political upheaval. They asked: Why this virus? Why now? That is, they felt driven to learn about the “configuration” of AIDS, emphasizing interconnection, system, and ecological balance (Rosenberg 1993; Anderson 2010). In contrast, most ordinary public health experts, along with the general public, continued to favour explanations affirming the dangers of “contamination” and social contact, the contagious aspects of disease transmission, those features that are both so exciting and so repellent. The default drive of conventional wisdom—especially in a perceived crisis—therefore was to “foreground a particular disordering element” (Rosenberg 1993, 295). Thus, we were easily distracted by prurient stories of “patient zero,” a French-Canadian airline steward, flying around merrily sowing the seeds of the pandemic (Auerbach et al. 1984; Shiltz 1987). Environmental, societal, and economic causes of the emergence of AIDS, and subsequent viral outbreaks, lacked the same charisma and glamour, the same ease of access. Until recently, most bioethicists seem to have been complicit in concentrating on sexy and vulgar contaminations rather than complex biosocial configurations. In part this tendency indexes the crisis framing, in which recourse to contamination becomes elementary and appealing; thus, it becomes necessary to think beyond or beside the “crisis” in order to feel the event’s configuration and to sense its process.

Reflecting on the AIDS pandemic, historian Charles E. Rosenberg (1998, 719) observed that epidemiological reasoning and medical practice are “at once occasions and rationales for culturally ubiquitous calls to reconsider and reorder modes of life.” At the end of the last century, perceptions of health and disease had come to be seen—by many elite biologically inclined microbiologists at least—in relation to “a more expansive global perspective in which inclusive and ecological styles of analysis have become increasingly pervasive” (726). According to Rosenberg, these ecological dimensions of the AIDS “crisis” were becoming evident as it appeared to fade. “An unceasing manipulator of the environment, humankind has never been able to attain a stable ecological relationship with the universe of potential pathogens” (727). Epidemiologists belatedly were coming around, becoming ever more ready to scrutinize ecologies of health and disease, often on a planetary scale. Yet until COVID-19, bioethicists generally hewed to a narrower path, continuing to decouple bios from biosphere.

## Making Ecological Sense of COVID-19

Ever since the emergence of the novel coronavirus in a Wuhan wet market late in 2019, ecological theories of the agent’s arrival and spread have contended with the conviction that humans must have made it and leaked it. To think of the virus as zoonotic, its presence reinforced and passage intensified through environmental disruption, commercial exploitation, and expanded communications and contact, has proven much harder than to imagine it as simply the result of contamination, the linear transmission from laboratory to market to the world. Despite accumulating evidence (Gao et al. 2022; Worobey et al. 2022; Pekar et al. 2022) that SARS-CoV-2 crossed over to humans from wild animals crowded into the Huanan market, some people insist that it must derive instead from a Chinese laboratory (Baker 2021). COVID-19 thus represents yet another case of humans trying to keep a virus, like everything else, to themselves. It demonstrates yet again the solipsism of popular epidemiology, the ingrained sense of human dominance and superior agency. And so, “lab zero” in the current pandemic has come to substitute for the patient zero of the AIDS pandemic. Continuing obsession with a possible Wuhan lab leak works to impede commitment to reasoning viral emergence ecologically (Anderson 2021b).^1^ For a time, even Jeremy Farrar (2021), the former director of the Wellcome Trust, entertained conspiracy theories about nefarious Chinese viral manipulation, presumably forgetting his past experiences studying natural outbreaks of dengue and avian influenza in Vietnam. In the United States, Anthony Fauci, former director of the National Institute of Allergy and Infectious Diseases, an estimable virologist with little comprehension of ecology, continues to argue that a laboratory leak, however unlikely, could possibly explain the origin of our current scourge (Wallace-Wells 2023).

Surprisingly, social theorists have occasionally shown better ecological intuition than many scientists during the pandemic. From the start, Bruno Latour recognized the pandemic as a manifestation of the global ecological crisis. For the French *grand penseur*, COVID-19 represented a “dress rehearsal” for other anticipated catastrophes of human-induced global heating (Latour 2021, 109). Thus, “the lockdown imposed by the virus could serve as a model for familiarizing us slowly with the general lockdown imposed by … the ‘environmental crisis’” (38). According to Latour, the pandemic shows us that we are no longer biopolitical individuals, but rather holobionts, entangled and overlapping with others, needing to find “new ways of placing ourselves differently in the same spot” (54). Slavoj Žižek soon amplified Latour’s concerns. The Slovenian philosopher saw that “the link between the Covid-19 pandemic and our ecological predicament is becoming ever more clear” (Žižek 2021, 26). He had learned from Latour and others that “epidemics erupt from our unbalanced relationship with our natural environs, they are not just a health problem” (71). It was obvious to him that “the Covid-19 pandemic announces a new epoch in which we will have to rethink everything, including the basic meaning of being human” (11)—including, one infers, what it means to do bioethics.

Feminist theorist Judith Butler also asks what kind of world is this in which such an event can happen—and how should we live in such a world? “How do these times and this world, already shifting in intensity, offer a chance to reflect upon interdependency, intertwinement, and porosity?” (Butler 2022, 34). She recognizes in a vague and indefinite way that the emergence of SARS-CoV-2 is related to climate change and environmental destruction. She understands that “an inhabitable world for humans depends on a flourishing earth that does not have humans at its center” (66). Ethical discourse must acknowledge the bonds we have with one another, with other species, and with the planet. Yet Butler seems unsure what to do with this insight. Similarly, Italian-Dutch philosopher Rosi Braidotti (2020, 465) asserts that

… the COVID-19 pandemic is a man-made disaster caused by undue interference in the ecological balance and lives of multiple species.” During the pandemic, “the power of viral relations has become manifest … stressing the agency of non-human forces and the overall importance of Gaia as a living symbiotic planet. (466)

Consequently, she demands that we “develop different ways of caring, a more transversal, relational ethics that encompasses the non-humans” (466)—and perhaps embraces the earth itself. Or as Žižek (2020, 475) puts it: “To confront the forthcoming ecological crisis, a radical philosophical change is … needed.” But just what might this entail?

## Bioethics to Earth

It may help to be reminded of the origin of the term “bioethics.” When Wisconsin biologist Van Rensselaer Potter came up with the locution in the early 1970s, he sought to connect ethical reasoning with evolutionary or ecological thought, distinguishing the subject from serviceable endeavours such as medical or professional ethics. His emphasis was on how to live well with the environment, how to inhabit a place responsibly, how to build a bridge to the future. An admirer of his Wisconsin colleague Aldo Leopold’s *Sand County Almanac* (1949) and geneticist C.H. Waddington’s *The Ethical Animal* (1960), Potter was eager to relate human health and well-being to a “land ethic,” linking humans to their ecological niches (ten Have 2019; Wardrope 2020).^2^ Drawing on systems ecology and cybernetics, Potter (1970, 243) imagined bioethics as stewardship of “the fragile web of non-human life that sustains human society.” Not long after the first Earth Day (which was instigated by Wisconsin senator Gaylord Nelson), Potter (1971, 179) implored Americans to “look upon earth, man, plants and animals, seas, and atmosphere as a balanced ecological system.”^3^ Human population health requires us to inhabit the earth conscientiously and properly, adapting sensitively to the biosphere, expressing “biophilia,” as E.O. Wilson (1986) proposed (see also Potter 1988). But as we know, this earth-bound Wisconsin vision of bioethics did not catch on—not even in Wisconsin.^4^ Soon it was displaced by more functionalist and narrowly conceived iterations of bioethics (Reich 1994)—or hijacked by alienated philosophers, as Potter lamented—a symptom of what neurologist Peter J. Whitehouse (1999, 41) called “the ecomedical disconnection syndrome.”

An alternative interpretation may be that Potter’s bioethics, like Leopold’s land ethic, was largely diverted in the 1970s into nascent environmental ethics, causing the half-constructed bridge between medical ethics and ecological reasoning to be abandoned. As it emerged after the 1972 Stockholm Conference on the Human Environment, environmental ethics tended to discard the anthropocentrism of bioethics. Focussed instead on structural critiques of capitalist exploitation, discussions of environmental stewardship became more and more isolated from bioethics inquiries as we have come to know them (Passmore 1974; Rolston 1988; Nash 1989). As environmental ethics developed in the late-twentieth century, it ramified into considerations of sustainability, conservation, animal rights, deep ecology, eco-Marxism, eco-feminism, and so on—but it scarcely touched on human population health, let alone clinical care and medical research practices. The rise of public health ethics in the twenty-first century drew increasing attention to issues of social justice and health equity, to the social determinants of health, to the role of public beneficence, to the need for social solidarity and connectedness (Dawson and Jennings 2012)—but rarely did public health ethics spotlight disease ecology and planetary health or extract value from environmental ethics.

But this simple dichotomy of medical ethics and environmental ethics has frequently been troubled during the past twenty years. From the margins, a dedicated band of ecologically minded ethicists and outsiders persisted in urging the necessary consilience of medical and environmental ethics, often couching such a course of action as the return to Potter’s original conception. In asking whether bioethics “can survive in a dying world,” for example, freelance ethicist Jessica Pierce (2002, 4) observed that “environmental thinking seems strangely isolated from the normal patterns of thought and discussion in bioethics.” Initially, she mostly was worried about the contributions of healthcare systems to environmental waste, air pollution, and carbon emissions, but over the years she came to fasten on the impact of degraded ecosystems on human population health. Pierce insisted that ethicists should explore “thinking based on the concept of connectedness and committed to viewing humans as not exempt from nature but part of it” (Pierce 2002, 5; see also Pierce and Jameton 2004). Similarly, pessimistic population biologist Paul R. Ehrlich (2009, 417) regretted that “bioethics does not provide much of an ethical base for considering human–nature relationships.” He suggested instead “ecoethics” as a means of holding “responsible those who are wrecking humanity’s life-support systems” (425). He remained especially concerned by unsustainable population growth. The “big ecoethical issue” for him was “how to reorganize global civilization ethically and consciously evolve its norms … so that it can transition to a sustainable and fair society” (427). At the time, most bioethicists did not share his sense of urgency (but see Resnick 2009; MacPherson 2013).

## Planetary Health Ethics?

Anxieties about climate change and the breakdown of the planet’s life-support systems became ever more pressing in the years leading up to the advent of the novel coronavirus. The development of One Health in the early 2000s (Woods et al. 2018; Anderson 2023) and the emergence of Planetary Health (Anderson and Dunk 2022) in the past decade have added impetus to moves toward reconciliation of bioethics and environmental ethics. One Health directed attention to the roles of non-human animals in the spread of infectious diseases, to zoonoses which often burst forth into epidemic outbreaks.^5^ While Planetary Health, systemically and at global scale, has emphasized the impact on human population health of the degradation of planetary ecosystems, principally through anthropogenic global heating, leading to extreme heat events, bushfires, drought, flooding, destruction of arable land, freshwater shortages, rising oceans, and the range expansion of vectors of infectious diseases. Tony L. Goldberg and Jonathan A. Patz (2015, 5, 6), influential advocates of Planetary Health, both at Wisconsin, posited a “global health ethic” premised on the “idea of health as an interconnected entity,” linking human health, animal health, and environmental health, and possessing the wherewithal to impel “sustained societal commitment” (see also Patz et al. 2007). In 2022, during the COVID-19 pandemic, the World Health Organization (2021) declared that climate change is “the biggest health threat facing humanity.” It would surely be lamentable, even untenable, if mainstream bioethics ignored the health implications and ethical correlates of destruction of the earth’s habitable spaces (ten Have 2019).

Despite the distraction of securing lab zero and the deficiencies of crisis framing, the appearance of the novel coronavirus has come to reinforce these proposals to ecologize bioethics. Thus, Peter Tagore Tan (2020, 53) argues that the inevitable “anthropocentrism of bioethics focuses too closely on the medical health of humans such that it paradoxically jeopardizes the very health of humans.” Instead, renewed and reinvigorated bioethics post-COVID “must be about the active role humans have in the ability of their place of habitation to heal itself” (60). Henk A.M.J. ten Have suggests that bioethics now needs to replace military metaphors based on fears of contagion and bio-invasion with more Hippocratic formulations figuring in cohabitation and natural balance. The pandemic, he writes, is “a consequence of the human way of life and exploitation of the planet” (ten Have 2020, 525). Therefore, we need “to develop practices based on relationality and connectedness … articulated in global bioethics with an ecological vocabulary” (527). After all, “human beings cannot be healthy when the planet is not healthy” (527). Lisa A. Eckenwiler (2020, 575) suggests “a global ecological ethic” that would re-imagine health “as interdependent and aim at ‘ethical placemaking’ across health ecosystems to enable people everywhere to have the capability to be healthy” (see also Lee 2017; Wardrope 2020). According to Andrew Jameton and Jessica Pierce (2021, 525), it is time for bioethics to recognize the obligation to support and protect nature—though it remains “unclear whether clinical bioethics can or will rise to the climate and ecological challenge.”

But there is something missing in this argument for the convergence of medical ethics and environmental ethics, something not quite captured in Potter’s original version of bioethics either. Our contemporary urgency in rendering bioethics fit for today’s ecological challenges has understandably directed us toward environmental and climate sciences and even into systems theories and cybernetics. But other sources of insight into habitability, or living well in place, often have been ignored or pre-empted. Instead, the turn to ecological bioethics should represent an opportunity to engage with Indigenous knowledge practices. It thus may be timely and ethical, as environmental humanities scholar Deborah Bird Rose (2005, 295) put it, to “re-situate the human in ecological terms … within an Indigenous philosophical ecology” (see also Johnston et al. 2007; Birch 2016; Whyte 2017; Redvers 2018; Pratt 2023). Indeed, I suspect that if Potter were contemplating bioethics today, he would be conversing with the Menominee people on whose land he dwelled. Maybe the pandemic will set us to listening to Indigenous knowledge leaders, belatedly.

At the turn of the last century. A.J. “Tony” McMichael (1999, 887), an environmental epidemiologist inclined to thinking on a global scale, a founder of Planetary Health, saw the need “to understand the determinants of population health beyond proximate, individual-level risk factors.” Facile assumptions of contamination and defilement, a linear contagion narrative, had been favoured over more complex ecological and sociological configurations of disease emergence and patterning. “Modern epidemiology’s search for specific proximate causes has deflected us from social-contextual models of disease causation,” McMichael wrote. “We epidemiologists must broaden our causal models and recognize the important ecologic dimensions of social-environmental influences on health and disease” (895–896). As the COVID-19 pandemic appears to wane, even as the conditions that gave rise to it continue to gather and intensify, it equally behoves bioethicists, as much as epidemiologists, to respond to McMichael’s plea. The pandemic might thus open a portal to another bioethics, just as AIDS once did. This requires us to return to Potter’s inspiration, his vision for a melded medical ethics and environmental ethics, if not to his actual theories and precepts—since both ethical reasoning and disease ecology have moved on and scaled up. Currently, most calls for renewed earth-bound bioethics or planetary health ethics are largely gestural, still not as close to the scalar complexities of contemporary ecological analysis as they should be (Buse, Smith, and Silva 2019). But that, too, will change. When it happens, we will see clearly that bioethicists all must supplement judgement with advocacy, campaigning not only for the health and dignity of the sick but also for the health and security of our plundered planet (Dunk et al. 2019; Williams et al. 2021). Let us hope that is one of the lessons of the COVID-19 pandemic.

## Footnotes

1 A similar argument can be made about preoccupations with vaccine development and other clinical framings.

2 “That land is a community is the basic concept of ecology,” Leopold (1949, viii–ix) wrote, “but that land is to be loved and respected is an extension of ethics.” It is likely Potter also was aware of the studies in medical geography conducted by another Wisconsin colleague, medical historian Erwin Ackerknecht (1965).

3 Potter’s ecological turn also coincided with the establishment of the U.S. Environment Protection Agency in 1970.

4 I recall that as chair of the Department of Medical History and Bioethics at Wisconsin, around 2005, I dismissed (following my bioethics colleagues’ advice) suggestions that we might revisit Potter’s work.

5 EcoHealth also emerged in the early 2000s, initially emphasizing the physical environment in distinction from One Health’s focus on interspecies disease transmission, but after a decade or so, it became effectively subsumed into One Health (Anderson and Dunk 2022).
